# Single-cell analysis reveals melanocytes may promote inflammation in chronic wounds through cathepsin G

**DOI:** 10.3389/fgene.2023.1072995

**Published:** 2023-01-23

**Authors:** Aobuliaximu Yakupu, Di Zhang, Haonan Guan, Minfei Jiang, Jiaoyun Dong, Yiwen Niu, Jiajun Tang, Yingkai Liu, Xian Ma, Shuliang Lu

**Affiliations:** ^1^ Department of Burn, Ruijin Hospital, Shanghai Jiaotong University School of Medicine, Shanghai, China; ^2^ Wound Healing Center, Ruijin Hospital, Shanghai Jiaotong University School of Medicine, Shanghai, China

**Keywords:** melanocytes, computational biology [mesh], wounds and injuries (MeSH), regeneration, cell communication

## Abstract

During acute wound (AW) healing, a series of proper communications will occur between different epidermal cells at precise temporal stages to restore the integrity of the skin. However, it is still unclear what variation happened in epidermal cell interaction in the chronic wound environment. To provide new insights into chronic wound healing, we reconstructed the variations in the epidermal cell-cell communication network that occur in chronic wound healing *via* single-cell RNA-seq (scRNA-seq) data analysis. We found that the intricate cellular and molecular interactions increased in pressure ulcer (PU) compared to AW, especially the PARs signaling pathways were significantly upregulated. It shows that the PARs signaling pathways’ main source was melanocytes and the CTSG-F2RL1 ligand-receptor pairs were its main contributor. Cathepsin G (CatG or CTSG) is a serine protease mainly with trypsin- and chymotrypsin-like specificity. It is synthesized and secreted by some immune or non-immune cells. Whereas, it has not been reported that melanocytes can synthesize and secrete the CTSG. F2R Like Trypsin Receptor 1 (F2RL1) is a member of proteinase-activated receptors (PARs) that are irreversibly activated by proteolytic cleavage and its stimulation can promote inflammation and inflammatory cell infiltration. In this study, we found that melanocytes increased in pressure ulcers, melanocytes can synthesize and secrete the CTSG and may promote inflammation in chronic wounds through CTSG-F2RL1 pairs, which may be a novel potential target and a therapeutic strategy in the treatment of chronic wounds.

## Introduction

Pressure ulcer (PU) is a type of chronic wound, and chronic wounds are characterized by a stalled undefined non-healing state, where dysregulated inflammation hinders the regeneration process, causing decreased angiogenesis, hyperproliferative non-migratory epithelium, dysregulated levels of cytokines/growth factors, and/or increased protease activity, and fibrosis (Frykberg and Banks, 2015; Martin and Nunan, 2015; Jones et al., 2018; Atkin, 2019; Las Heras et al., 2020; Sawaya et al., 2020). Wound healing is a complex, highly regulated process that involves interactions between various cell types. Epidermal cells activate at precise temporal stages and properly communicate with immune or non-immune cells during acute wound (AW) healing (Tobin, 2006; Martin and Nunan, 2015; Rodrigues et al., 2019). However, how the epidermal cells interact with each other in the chronic wound environment is still unclear.

Cathepsin G (CatG or CTSG) is a serine protease mainly with trypsin- and chymotrypsin-like specificity (Avril et al., 1994; Thorpe et al., 2018). It is expressed by neutrophils, mast cells, primary monocytes, and professional antigen-presenting cells (B cells, conventional dendritic cells (DC), plasmacytoid DC, and murine microglia) (Schechter et al., 1994; Yager and Nwomeh, 1999; Greener et al., 2005; Edwards et al., 2007; Korkmaz et al., 2008; Stoeckle et al., 2009; Burster et al., 2010). Additionally, CTSG is also found in some non-immune cells, such as endothelial and smooth muscle cells (Wang et al., 2014), brain astrocytes (Abraham et al., 1993), fibroblasts (Cavarra et al., 2002), and in Paneth cells–specialized epithelial cells underneath the crypts of Lieberkühn (Zamolodchikova et al., 2017; Zamolodchikova et al., 2020), whereas it has not reported that melanocytes can synthesize and secrete the CTSG. F2R Like Trypsin Receptor 1 (F2RL1) is a member of proteinase-activated receptors (PARs) that are irreversibly activated by proteolytic cleavage and its stimulation can promote inflammation, inflammatory cell infiltration (Miike et al., 2001; Shpacovitch et al., 2004; Shpacovitch et al., 2007; Cheng et al., 2017; Gao et al., 2017). In wound healing, melanocytes were found to undergo mitosis in the adjacent uninjured skin and are involved in regulating keratinocyte differentiation (Hirobe, 1983; Paus, 2013), while It has not been found that melanocytes can regulate inflammation.

In this study, we reconstructed the cell-cell communication network *via* single-cell RNA-seq (scRNA-seq) and found that cellular and molecular interactions of epidermal cells are enhanced in pressure ulcers, melanocytes can synthesize and secrete the CTSG and may promote inflammation in chronic wounds through CTSG-F2RL1 pairs.

## Material and methods

### scRNA-seq data and analysis workflow

#### Quality control of scRNA-seq data

The single-cells isolated for sequencing from intact skin (n = 4), AW (n = 4), and PU(n = 5), all nine donors are males with an average age of 37.6 (the average age of donors who donated intact skin: 29.8; AW: 29.5; PU: 44) (Supplementary Table 1). We retrieved the filtered read count matrix of 1170 cells that passed the stringent quality control in the original article (including 1) expression of more than 1000 genes with RPKM >1; 2) total read counts >50K; 3) uniquely mapping ratio >0.4; 4) Spearman correlation coefficients between any two nearest cells >0.4.1170 out of 1511 cells passed these four criteria.) from the uninjured skin and wound epidermis from the GEO (GSE137897) (Li et al., 2021).

#### Unsupervised identification of epidermal cell clusters

We next used the count matrix from three different conditions [normal skin (NS, n = 391), acute wound (AW, n = 398), and pressure ulcer (PU, n = 381)] to create three different Seurat object with the R package “Seurat” (Version 4.0.4), which can exert a comparative analysis by heterogeneous tissues across different conditions (Hao et al., 2021). Then, we performed Normalizing, PCA, and determine the ‘dimensionality’ of the dataset by JackStraw and Elbow analysis. The cells from three different conditions were projected to the t-distributed Stochastic Neighbor Embedding (t-SNE) dimensions by the first 15 PCs (Hao et al., 2021). We identified 6 cell clusters at a resolution of 0.8 (function “FindClusters”), and the number of clusters at different clustering resolutions as shown in Supplementary Figures S1A–S1C) using the R package “clustree” (Version 0.5.0). We selected the resolution of 0.8 after comparing the number of clusters in different resolutions in three sample types. In each sample type, we calculated the differentially expressed genes (DEGs) for individual cell clusters compared to all other cluster cells using the method of Likelihood-ratio test for single-cell gene expression (function “FindMarkers” with min pct = 0.25). The DEGs were expressed in the respective cluster with a log2 (fold-change)≥1 and adjusted *p*-value <0.05 Cell types of each cluster were identified according to canonical, novel markers revealed by differential expression analysis and KEGG/GO analysis.

### Gene ontology (GO) and kyoto encyclopedia of genes and genomes (KEGG) analysis

GO analysis was performed for the differentially expressed genes (DEGs) with Enrichr by R package “Cluster Profiler” (Version 4.0.5) (Chen et al., 2013; Kuleshov et al., 2016). Among the GO biological process terms with *p*≤0.01 and KEGG terms with *p*≤0.05.

### Inference and analysis of cell-cell communication

#### CellChat analysis of each dataset from AW and PU

We separately created the CellChat object with the R package “CellChat” (Version 1.1.3) for AW and PU datasets by their annotated Seurat object. We reconstructed the cell-cell autocrine and paracrine signaling interactions through CellChatDB after setting for humans. CellChatDB is a manually curated database (Li et al., 2021). Then, we compute the communication probability by integrating gene expression per cell group and filtering out the cell-cell communication if there are fewer than 10 cells in certain cell groups. CellChat infers cell-cell communication at a signaling pathway level (Jin et al., 2021). We calculate and visualized the aggregated cell-cell communication network with default parameter; Computed the contribution of each ligand-receptor pair to the specific signaling pathway and visualized cell-cell communication mediated by multiple ligand-receptor pairs; Plot the gene expression distribution of signaling genes related to specific signaling pathway; We also identified signaling roles (e.g., dominant senders, receivers) of cell groups as well as the major contributing signaling, and identify signals contributing most to outgoing or incoming signaling of certain cell groups with default parameter (https://htmlpreview.github.io/?https://github.com/sqjin/CellChat/blob/master/tutorial/CellChat-vignette.html) (Jin et al., 2021).

#### CellChat comparison analysis between AW and PU

To identify the conserved and context-specific signaling pathways in different wound healing conditions, we further operated a comparative analysis between AW and PU by CellChat. First, we merged the different CellChat objects (AW and PU). Then, we compared the total number of interactions and interaction strength. Further calculated the differential number of interactions and interaction strength among different cell types. To simplify the complicated network and gain insights into the cell-cell communication at the cell type level, we aggregated the cell-cell communication based on the defined cell groups (aggregated basal keratinocyte clusters, spinous keratinocyte cluster to keratinocytes group, melanocyte clusters to melanocytes group, immune cell clusters to immune cells group and “mitotic” clusters to “mitotic” cells group). Then, we identified and visualized the conserved and context-specific signaling pathways and corresponding upregulated and downregulated signaling ligand-receptor pairs (https://htmlpreview.github.io/?https://github.com/sqjin/CellChat/blob/master/tutorial/Comparison_analysis_of_multiple_datasets.html) (Jin et al., 2021). All codes along with input and output data used during the current study are available in the Github repository, https://github.com/Haximm/cell.

### Human wound samples

For the validation has strong persuasiveness, the condition of the tissue samples used for the validation of the bioinformatic findings were fairly correspondence with tissue samples used for scRNA-seq (GSE137897) (Supplementary Table S2). Tissue samples were taken from acute wounds and pressure ulcers in patients attending the Wound Healing Center, Ruijin Hospital, Shanghai Jiaotong University School of Medicine, Shanghai 200,025, China, with informed consent. Ethical approval for the study was given by the Ethics Committee of the Ruijin Hospital, Shanghai Jiao Tong University School of Medicine 200,025, China [(2018) NLS No. (27)]. The Tissue samples were fixed overnight in 10% formalin (Brv-0020–0010, Shanghai Runnerbio Technology CO. Ltd., China), dehydrated sequentially with ethanol, and subsequently embedded in paraffin (Shanghai Runnerbio Technology CO. Ltd., China).

### Immunohistochemical staining

Immunohistochemical staining was performed on deparaffinized, formalin-fixed consecutive tissue sections (3–4 μm thickness) with PMEL (ab137078, Abcam, UK), CTSG (ab197354, Abcam, UK), and F2RL1 (sc-13504, Santa Cruz Biotechnology, United States).

### Immunofluorescence

After deparaffinization and rehydration, consecutive sections (3–4 μm thickness) were treated for antigen retrieval, block with serum and incubated overnight at 4°C with primary antibody for PMEL (ab137078, Abcam, UK), CTSG (ab197354, Abcam, UK), and F2RL1 (sc-13504, Santa Cruz Biotechnology, United States) at a dilution of 1:100 (CTSG, ab197354; F2RL1, sc-13504) and 1:200 (PMEL, ab137078). The next days, the sections were incubated with the secondary antibodies and cell nuclei were stained with 4,6-diamidino-2-phenylindole (DAPI) (Supplementary Table S3). Microscope slides were converted to digital slides using a digital microscope equipped with a slide scanner system (PannoramicMIDI; 3DHISTECH, Hungary).

### Evaluation of immunohistochemical staining and statistical analysis

All slides were independently scored by three researchers who were blinded to the clinical parameters of patients. The staining intensity was scored on the following scale: 0, no staining; 1, weak staining; 2, moderate staining; and 3, intense staining. Results from three researchers were averaged to arrive at a single final score and used in the statistical analysis. All data were expressed as mean ± SD and plotted using GraphPad Prism, version 8.0.1 (GraphPad Software, San Diego, CA). Statistical significance was determined by Mann‒Whitney Test. For all statistical tests, *p* < 0.05 was considered to be statistically significant. *p < 0.05, **p < 0.01.

## Results

### Classification of epidermal cell constitution of uninjured skin and wounds

**FIGURE 1:**
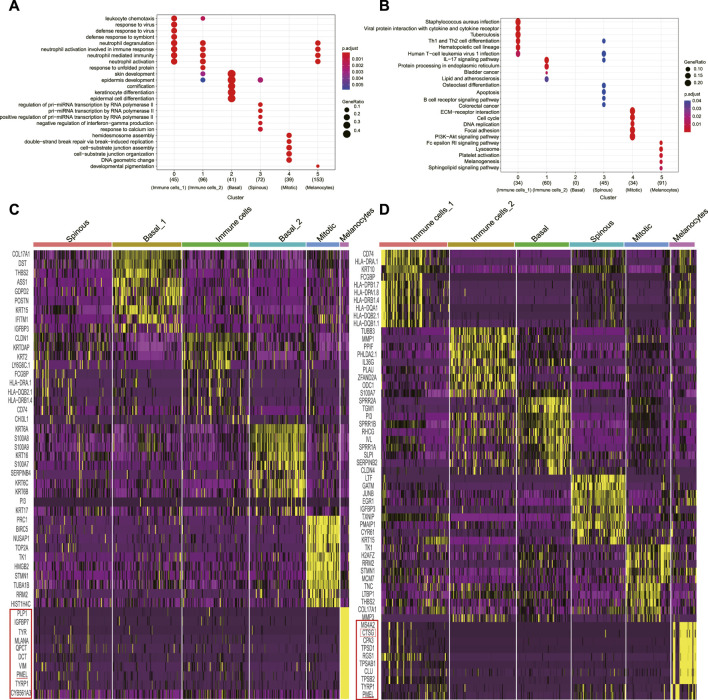
Classification of uninjured skin and wound epidermal cell constitution. **(A)**, GO analyses of each cluster for cluster annotation in PU. **(B)**, KEGG analyses of each cluster for cluster annotation in PU. **(C)**, Heatmap of each cell population’s top 10 genes’ expression to other cell populations (yellow) in AW, some genes are not shown. **(D)**, Heatmap of each cell population’s top 10 genes’ expression to other cell populations (yellow) in PU, some genes are not shown. AW: acute wounds; PU: pressure ulcers.

We retrieved and analyzed the filtered read count matrix of 1170 cells from three different conditions, i.e., NS (n = 391), AW (n = 398), and PU (n = 381) from the GEO (GSE137897) (Li et al., 2021). These epidermal cells were separated into six clusters each by Seurat clustering (resolutions = 0.8) (Supplementary Figures S1A–S1C). The annotation based on that basal layer keratinocyte had the highest expression levels of KRT5 and KRT14; spinous keratinocyte displayed high DSG1 and DSP levels with the highest expression of KRT1 and KRT10; granular keratinocyte expressed a suite of late differentiation markers, including LOR, FLG, and SPINK5; and melanocytes had a higher expression of PMEL, TYRP1, and MLANA, whereas immune cells had higher levels of CD74 (Supplementary Figures S2A–S2C). Combining canonical, novel markers in differential analysis and KEGG/GO analysis allowed us to annotate different clusters (Figures 1A, B, Supplementary Figure S1D, E, Supplementary Figures S3–S5) (Cheng et al., 2018; Finnegan et al., 2019; Sanz-Gómez et al., 2020; Wang et al., 2020; Enzo et al., 2021).

In uninjured skin, we annotated one basal (n = 106), granular (n = 74), and spinous (n = 74) keratinocyte cluster, one cluster named melanocytes (n = 65), one for immune cell (n = 58) and one termed as “mitotic” cell (n = 14) because of the high level of well-recognized DNA synthesis and cell division transcripts, such as PCNA and KI67 (Supplementary Figure S1F, Supplementary Figure S6A).

Following the same criterion, we annotated two basal (n = 88 and n = 72) keratinocyte clusters, one spinous (n = 100) keratinocyte cluster, one melanocytes cluster (n = 11), one immune cell cluster (n = 85) and one “mitotic” cluster (n = 42) in AW (Figure 1C, Supplementary Figure S6B). In PU, we annotated one basal (n = 66) and one spinous (n = 66) keratinocyte cluster respectively, one melanocyte (n = 31) cluster, two immune cell clusters (n = 83 and n = 81), and a “mitotic” cluster (n = 54) (Figure 1D, Supplementary Figure S6C).

### Cellular and molecular interactions of epidermal cells are enhanced in pressure ulcers

**FIGURE 2:**
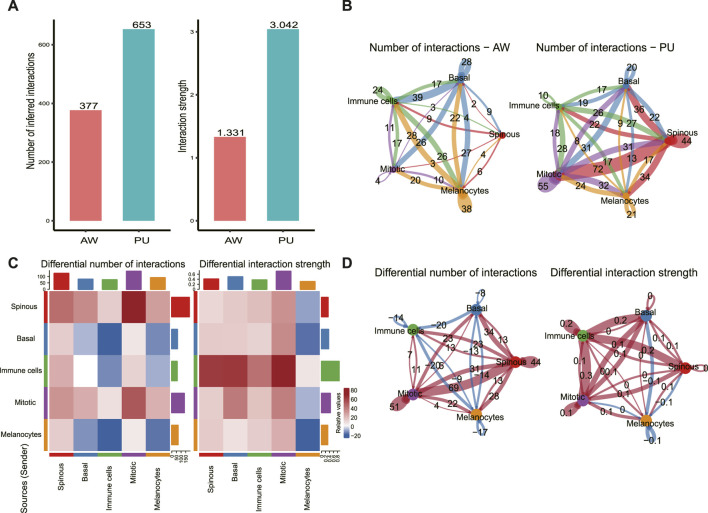
Cellular and molecular interactions of epidermal cells are enhanced in chronic wounds. **(A)**, Comparison of the total number of interactions and interaction strength; cell-cell communication is enhanced in chronic wounds. **(B)**, The number of interactions between any 2 cell populations in acute wounds (AW) and pressure ulcers (PU). **(C)**, The differential number of interactions and interaction strength in the cell-cell communication network between AW and PU. The top colored bar plot represents the sum of the column of values displayed in the heatmap (incoming signals). The right colored bar plot represents the sum of the row of values (outgoing signals). In the color bar, red (or blue) represents increased (or decreased) signaling in the PU compared to AW. **(D)**, The differential number of interactions and interaction strength between any 2 cell types. Red (or blue) colored edges represent increased (or decreased) signaling in the PU compared to the AW: acute wounds; PU: pressure ulcers.

We used CellChat to predict the general principles of cell-cell communication. Comparing the total number of interactions and interaction strength, we found that the number and strength of cell-cell communication were enhanced in PU conditions (Figure 2A). In AW, the highest number of interactions was found between basal keratinocytes with other cells (92), followed by the melanocytes with other cells (70) (Figure 2B). In PU, the highest number of interactions was found between spinous keratinocytes with other cells (106) (Figure 2B). Furthermore, we compared the number of interactions and interaction strength among different cell populations to identify those which showed significant changes. The results show that the number of interactions between spinous keratinocytes and “Mitotic” cells increased significantly, while the strength of interactions increased most between immune cells and “Mitotic” cells (Figure 2C, D).

### PARs signaling pathway was significantly changed in pressure ulcers

**FIGURE 3:**
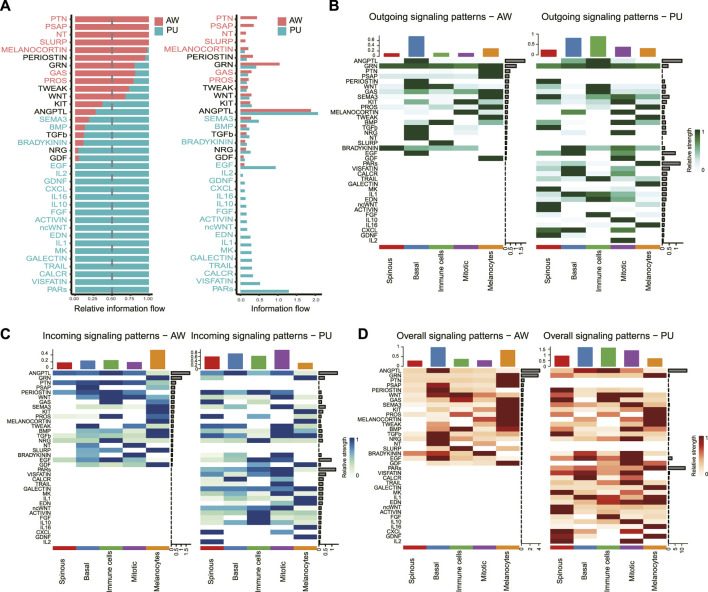
PARs signaling pathway was significantly changed in pressure ulcers. **(A)**, Significant signaling pathways were ranked based on differences in the overall information flow within the inferred networks between AW and PU. The signaling pathways depicted in red are enriched in AW, and those depicted in green were enriched in PU. **(B–D)**, Outgoing signals, incoming signals, and overall signaling by aggregating outgoing and incoming signaling together. AW: acute wounds; PU: pressure ulcers.

We identified the significantly altered signaling pathways by simply comparing the information flow for each, which is defined by the sum of communication probabilities among all pairs of cell groups in the inferred network (i.e., the total weights in the network). Significantly changed signaling pathways were ranked based on differences in the overall information flow within the inferred networks between the AW and PU. The results show the signaling pathway with the strongest variation in PU was the protease-activated receptors (PARs) signaling pathway (Figure 3A).

We further analyzed the outgoing and incoming signaling associated with each cell population between AW and PU. From the perspective of each cell, the results show that the highest outgoing signals were found in the basal keratinocytes in AW, and the immune cells in PU (Figure 3B). However, the highest incoming signals appeared in the melanocytes in AW, the “Mitotic” cells in PU (Figure 3C). In addition, the highest overall signals that combining of outgoing and incoming signals were found in the basal keratinocytes both in AW and PU conditions (Figure 3D). From the perspective of each signaling pathway, the result shows that although the ANGPTL signaling is the highest incoming, outgoing, and overall signal (combining of outgoing and incoming signals) both in AW and PU, the PARs signaling pathway was the most altered incoming, outgoing and overall signal (combining of outgoing and incoming signals) between AW and PU (Figures 3B–D).

### The melanocyte cell population was the dominant sender of the PARs signaling pathway

**FIGURE 4:**
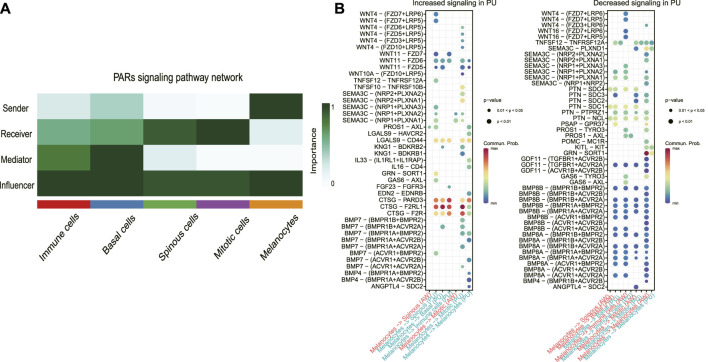
The melanocyte cell population was the dominant sender of the PARs signaling pathway. **(A)**, Dominant senders, receivers, mediators, and influencers of PARs signaling pathways. **(B)**, the upregulated (increased) and downregulated (decreased) signaling ligand-receptor pairs in PU compared to AW. The increased signaling means these pathways have a higher communication probability (strength) in PU compared to AW. AW: acute wounds; PU: pressure ulcers.

To define the signaling sources most subject to variation, we identified the dominant senders, receivers, mediators, and influencers in the substantially altered pathways. In the PARs signaling pathway at PU condition, the major senders are the melanocytes, whereas the spinous and mitotic cells are the main receivers and accept almost all the other cells’ influence (Figure 4A). The significantly increased PARs signaling pathway from the melanocytes indicates that melanocytes may play a crucial role in the healing process.

Next, we identified the upregulated and downregulated signaling ligand-receptor pairs in PU and determined the communication probabilities mediated by ligand-receptor pairs from melanocytes to other cell groups (Figure 4B). It showed that the highest communication probabilities mediated by ligand-receptor pairs from melanocytes compared to other cell groups are CTSG-F2RL1, CTSG-PARD3, and CTSG-F2R, which substantially increased in PU.

### Melanocytes may lead to chronic inflammation in pressure ulcers through CTSG

**FIGURE 5:**
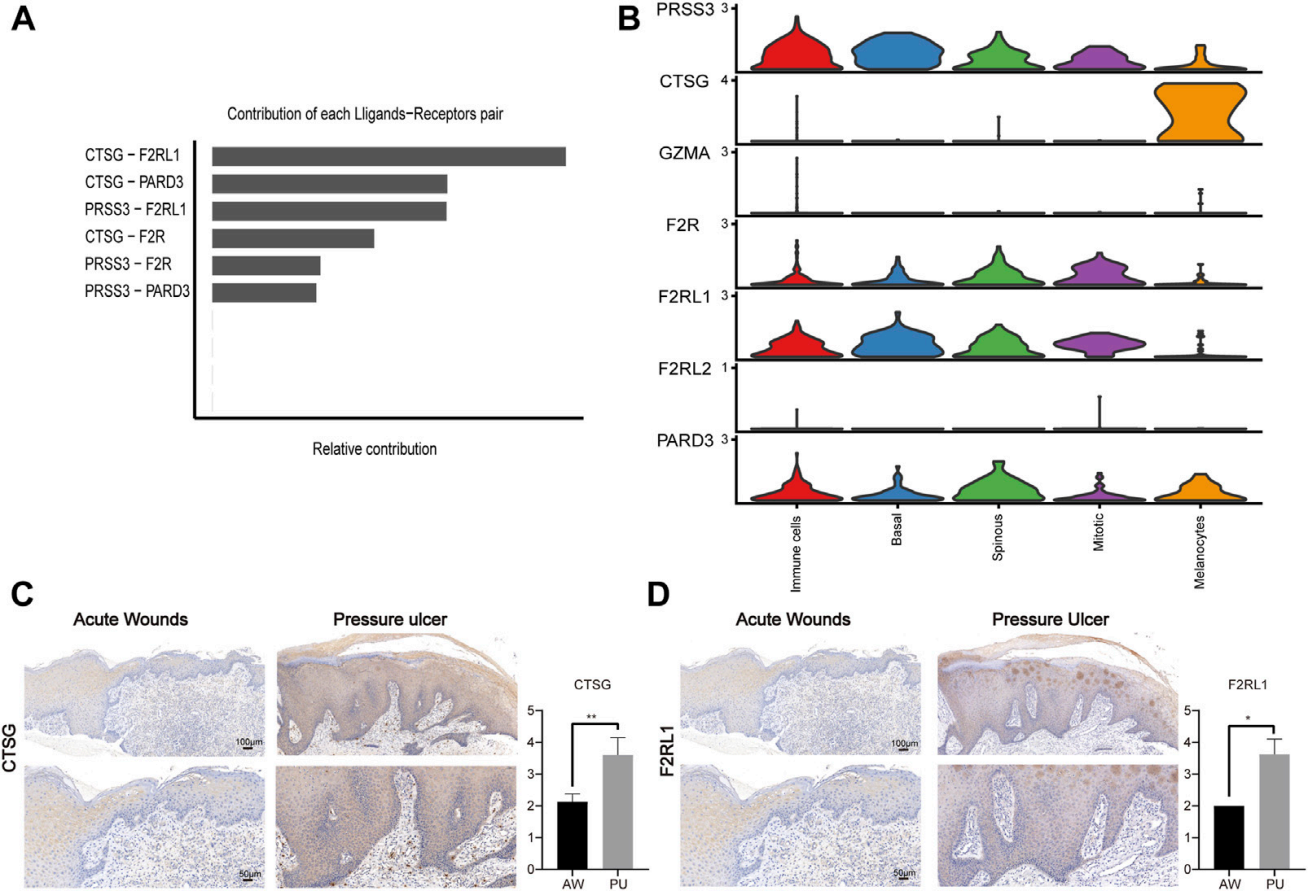
The CTSG and F2RL1 expressions increased in PU. **(A)**, The contribution of each ligand-receptor pair in the PARs pathway in PU. **(B)**, the expression of the genes related to the PARs pathway. **(C)**, the immunohistochemical staining results of CTSG in AW (n = 4) and PU (n = 5) tissue. Data represent the mean ± S.D. **p < 0.01. (Scale bar: 100, 50 μm). **(D)**, the immunohistochemical staining results of F2RL1 in AW (n = 4) and PU (n = 5) tissue. Data represent the mean ± S.D. *p < 0.05. (Scale bar: 100, 50 μm). AW: acute wounds; PU: pressure ulcers.

PARs signaling pathway was significantly increased in PU and the ligand-receptor pair in the PARs pathway that contributes most to cell-cell communication is CTSG-F2RL1 (Figure 5A). Through bioinformatics analysis, we found that CTSG has the highest expression in the melanocyte population of PU and F2RL1 has high expression in all cells except melanocytes (Figure 5B, Supplementary Figure S7A–S7D). We further validated our bioinformatics findings with tissue samples by immunohistochemical staining, the results show that the CTSG and F2RL1 expressions increased in PU compared to AW (Figures 5C, D).

**FIGURE 6:**
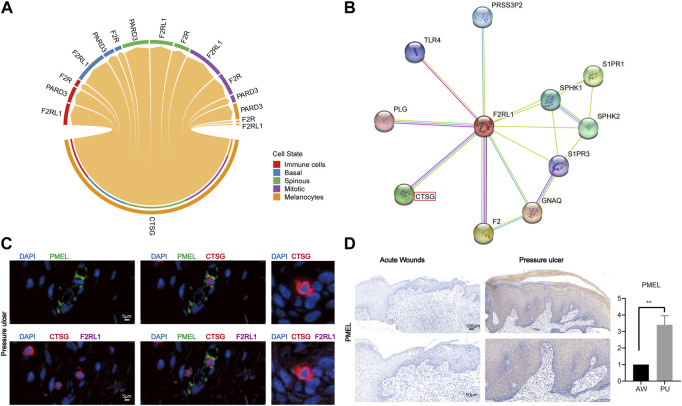
Melanocytes may lead to chronic inflammation in pressure ulcers through CTSG. **(A)**, The cell-cell communications of melanocytes population with other cell populations *via* ligand-receptor pairs of PARs pathway in PU. **(B)**, STRING interaction network of CTSG and F2RL1 protein. **(C)**, the immunofluorescence results of PMEL, CTSG, and F2RL1 in PU tissue (n = 4, Scale: 5 μm). **(D)**, the immunohistochemical staining results of PMEL in AW (n = 4) and PU (n = 5) tissue. Data represent the mean ± S.D. **p < 0.01 (Scale bar: 100, 50 μm). AW: acute wounds; PU: pressure ulcers.

Previous studies show that CTSG, as a serine protease, can interact with F2RL1 which is a member of PARs (Wiedow and Meyer-Hoffert, 2005; Pham, 2006). Our analysis shows that the CTSG from melanocytes can interact with the F2RL1 in other cells (Figure 6A), their protein interaction also can be found in the STRING interaction network (Figure 6B). The immunofluorescence results show that melanocytes indeed can express and secrete the CTSG, which can bind to other epithelial cells’ F2RL1 receptors in PU conditions (Figure 6C, Supplementary Figure S8). In addition, we found that melanocytes increased in PU compared to AW (Figure 6D).

## Discussion

PU are chronic lesions, localized injuries to the skin and underlying soft tissue caused by pressure, shear, friction, or a combination of these, often present in patients with limited mobility or sensory perception and paralyzed or unconscious patients who can neither sense nor reply to the intermittent need for changing the position (Dam et al., 2011; Zhao et al., 2016; Zhao et al., 2017; Mervis and Phillips, 2019). Although many advanced therapies are available for chronic wounds, such as growth factor-based therapies, negative pressure, oxygen, shock wave, and photobiomodulation therapies, each therapy presents limited benefits, and clinical outcomes vary among patients (Han and Ceilley, 2017; Jones et al., 2018; Oliveira et al., 2020). This study aims to identify the variation in cell communication to provide a deeper understanding of PU pathophysiology and novel promising therapeutic targets for PU.

In the epidermis, the keratinocytes are arranged in four layers to protect subcutaneous tissues and are in contact with melanocytes, dendritic, neural crest-derived cells that synthesize melanosomes (Hearing and Hori, 1997; Costin and Hearing, 2007; Rodrigues et al., 2019); Immune cells participate in skin adaptive and innate immunity, regulate every stage of wound healing *via* the secretion of various cytokines, chemokines, and growth factors (Gurtner et al., 2008; Lowes et al., 2014; Pasparakis et al., 2014; Kabashima et al., 2019). During acute wound healing, epidermal cells will activate at precise temporal stages, and proper communication will occur between resident or recruited, immune or non-immune cells (Martin and Nunan, 2015; Rodrigues et al., 2019), the study shows that basal keratinocytes have the highest interactions with other cells during AW. We found that the intricate cellular and molecular interactions increased in PU compared to AW, especially the communication between spinous and mitotic cell populations was increased significantly and the immune cells were the major signal sources and has the highest increased interaction strength in PU condition, it may indicate that the chronic inflammation states in PU. In addition, we found that the PARs signaling pathways show the strongest variation in the PU condition. PARs are G-protein-coupled receptors, activated by cleavage of their N-terminal domains by serine proteases, which are seven-transmembrane domain receptors also comprising PAR1, 2(F2RL1), 3, and 4 (Coelho et al., 2003). PARs are involved in the regulation of cardiac, skin, arthritis, and respiratory system physiological and pathophysiological functions and are the target of therapeutic drugs (Schmidlin et al., 2002; Gieseler et al., 2013; Oikonomopoulou et al., 2018; Carroll et al., 2021; Peach et al., 2023).

We found that the CTSG-F2RL1 ligand-receptor pairs were the main contributor to PARs signaling in PU. CTSG is a key serine protease, that can be found in a variety of cells, and it has both pro-inflammatory and anti-inflammatory activity including regulatory, bactericidal, and destructive functions, which depend on the physiological conditions (Zamolodchikova et al., 2020). The biological functions of CTSG are also relevant to their sources, the functions of CTSG are described as degradation of ECM components, plasma proteins, bactericidal properties, cleavage of receptors and inflammatory mediators, conversion of angiotensin I to angiotensin II, and platelet activation from neutrophils and monocyte (Korkmaz et al., 2008), antigen processing and presentation from antigen-presenting cells (Stoeckle et al., 2009; Burster et al., 2010), promote or suppresses atherosclerosis from endothelial and smooth muscle cells (Wang et al., 2014), assists in early wound repair in the brain from astrocytes (Abraham et al., 1993), contribute to the remodeling of sun-damaged skin from fibroblasts (Cavarra et al., 2002), antibacterial protection of epithelial cells from Paneth cells (Zamolodchikova et al., 2017; Zamolodchikova et al., 2020).

As a member of PARs, F2RL1 is widely expressed by cells, such as fibroblasts, neurons, vascular smooth muscle cells, and keratinocytes (Chandrabalan and Ramachandran, 2021). F2RL1 is cleaved and activated by a variety of endogenous serine proteinases with trypsin-like specificity, including coagulation factors VIIa, tissue factor (TF), trypsin, kallikrein, cathepsins, and induces different transduction pathways depending on the activating protease (Rattenholl and Steinhoff, 2008; McIntosh et al., 2020; Bang et al., 2021). It has been reported that F2RL1 is well expressed in the skin epidermis and implicated in the regulation of keratinocyte proliferation/differentiation, maintenance of the epidermal barrier, inflammation, and pruritus (Hussain et al., 2019; McIntosh et al., 2020). CTSG has in primates both chymase and tryptase activity and the chymase/tryptase activates the F2RL1 by cleaving its tethered ligand (Gao et al., 2017; Hellman et al., 2022; Redhu et al., 2022). However, it is reported that CTSG from neutrophils disarms the F2RL1 signaling (Dulon et al., 2003; Chin et al., 2008; Ramachandran et al., 2011). It also reported that serum CTSG induced the expression of F2RL1 and altered the dermal microvascular endothelial cells permeability to increase the inflammatory cells infiltration (Gao et al., 2017), but there is no clear demonstration of whether the CTSG from other cells active or disarm the F2RL1 signaling. It indicates that CTSG is responsible for activating or disarming the F2RL1 signaling in many sources that remain to be determined. However, previous studies have found that CTSG promotes inflammation (Lomas et al., 1995; Sun et al., 2004; Shimoda et al., 2007; Liu et al., 2015), while F2RL1 is well established as a driver of inflammatory responses in the skin (Steinhoff et al., 2005; Chandrabalan and Ramachandran, 2021; Fleischer et al., 2022). Therefore, we deduced that the CTSG proteolytic cleavage of the F2RL1 tethered ligand may promote inflammation during chronic wound healing.

We found that the PARs signaling pathways’ main source was melanocytes in the PU environment. The role of melanocytes in wound healing was first observed in the 1950s when it was reported that melanocytes appeared in the new epithelial covering within 4–6 days following the lesion (Snell, 1963). Previous studies reported that melanocytes were found to undergo mitosis in the adjacent uninjured skin and are involved in regulating keratinocyte differentiation (Hirobe, 1983; Paus, 2013; Yuan and Jin, 2018). This study shows that melanocytes were increased in PU compared to AW. In addition, the CTSG expression was increased and it was mostly expressed by melanocytes in the PU environment. The connection of CTSG with F2RL1 may promote inflammation, coupled with the melanocytes having the highest expression of CTSG and being the main source of the PARs pathway, which indicates that melanocytes may contribute to chronic inflammation in PU.

## Conclusion

Melanocytes not only can synthesize and secrete the CTSG but also has the highest expression of CTSG in the PU condition. Melanocytes may increase inflammation through CTSG-F2RL1 pairs in PU. It shows the melanocytes’ newest role in wound healing and CTSG-F2RL1 pairs may be a novel potential target and a therapeutic strategy in the treatment of chronic wounds.

## Data Availability

Publicly available datasets were analyzed in this study. This data can be found here: The datasets generated and/or analyzed during the current study are available in the GEO repository (GSE137897), https://www.ncbi.nlm.nih.gov/geo/query/acc.cgi?acc&equals;GSE137897. All codes along with input and output data used during the current study are available in the Github repository, https://github.com/Haximm/cell.
