# Supplementary material for: Single-cell analysis reveals melanocytes may promote inflammation in chronic wounds through cathepsin G
Source: Front Genet. 2023 Jan 23;14:1072995. doi: 10.3389/fgene.2023.1072995 (PMC9900029; doi:10.3389/fgene.2023.1072995)
Supplement: Supplementary file 1 [file Table1.docx]

**Single-cell analysis reveals melanocytes may promote inflammation in chronic wounds through Cathepsin G**

Aobuliaximu Yakupu ^1,2*^, Di Zhang^1,2*^, Haonan Guan^1,2^, Minfei Jiang^1,2^, Jiaoyun Dong^1,2^, Yiwen Niu^1,2^, Jiajun Tang^1,2^, Yingkai Liu^1,2^, Xian Ma^1,2 #^ and Shuliang Lu^1,2 #^

**Supplementary Figure legends:**

**Supplementary Figure S1.** **Classification of uninjured skin and wound epidermal cell constitution. (A),** the number of clusters in different resolutions in NS (the RNA_snn_res stands for the resolution parameter). **(B),** the number of clusters in different resolutions in AW (the RNA_snn_res stands for the resolution parameter). **(C),** the number of clusters in different resolutions in PU (the RNA_snn_res stands for the resolution parameter). **(D),** GO analyses of each cluster for cluster annotation in AW. **(E),** KEGG analyses of each cluster for cluster annotation in AW. **(F),** Heatmap of each cell population's top 10 genes’ expression to other cell populations(yellow) in PU, some genes are not shown. NS: Normal skin; AW: acute wounds; PU: pressure ulcers.

**Supplementary Figure S2.** **Annotation of uninjured skin and wound epidermal cell clusters. (A),** the expression of canonical markers of cells in different clusters in NS. **(B),** the expression of canonical markers of cells in different clusters in AW. **(C),** the expression of canonical markers of cells in different clusters in PU. NS: Normal skin; AW: acute wounds; PU: pressure ulcers.

**Supplementary Figure S3. Annotation of epidermal cell clusters in uninjured skin.**

**(A-F),** GO analyses of each cluster (0-5) for cluster annotation in NS. **(G-L),** KEGG analyses of each cluster (0-5) for cluster annotation in NS. NS: Normal skin.

**Supplementary Figure S4.** **Annotation of epidermal cell clusters in AW.**

**(A-F),** GO analyses of each cluster (0-5) for cluster annotation in AW. **(G-L),** KEGG analyses of each cluster (0-5) for cluster annotation in AW. AW: acute wounds.

**Supplementary Figure S5.** **Annotation of epidermal cell clusters in PU.**

**(A-F),** GO analyses of each cluster (0-5) for cluster annotation in PU. **(G-L),** KEGG analyses of each cluster (0-5) for cluster annotation in PU. PU: pressure ulcers.

**Supplementary Figure S6.** **Classification and annotation of uninjured skin and wound epidermal cell constitution. (A),** the t-SNE result of cells from NS. **(B),** the t-SNE result of cells from AW. **(C),** the t-SNE result of cells from PU. KC: keratinocyte; NS: Normal skin; AW: acute wounds; PU: pressure ulcers; t-SNE: t-distributed Stochastic Neighbor Embedding.

**Supplementary Figure S7.** **The CTSG and F2RL1 expressions increased in PU. (A),** the comparison of CTSG gene expression between NS, AW, and PU using scRNA-seq data (GSE137897). **(B),** the comparison of F2RL1 gene expression between NS, AW, and PU using scRNA-seq data (GSE137897). **(C),** the comparison of CTSG gene expression in each type of cell between NS, AW, and PU using scRNA-seq data (GSE137897). **(D),** the comparison of F2RL1 gene expression in each type of cell between NS, AW, and PU using scRNA-seq data (GSE137897). AW: acute wounds; PU: pressure ulcers; sc-RNA-seq: single-cell RNA-seq. (the three picture was drawn automatically online, http://130.229.28.87/shiny/rna_app/ )

**Supplementary Figure S8.** **Melanocytes may lead to chronic inflammation in pressure ulcers through CTSG.**

the immunofluorescence results of PMEL, CTSG, and F2RL1 in PU tissue (n=4, Vertical arrows indicate the secreted CTSG by melanocyte, and Transverse arrows indicate CTSG in melanocyte; Scale bar: 50μm, 5μm, 2μm).
