# Supplementary figures and images for: Single-cell analysis reveals melanocytes may promote inflammation in chronic wounds through cathepsin G
Source: Front Genet. 2023 Jan 23;14:1072995. doi: 10.3389/fgene.2023.1072995 (PMC9900029; doi:10.3389/fgene.2023.1072995)

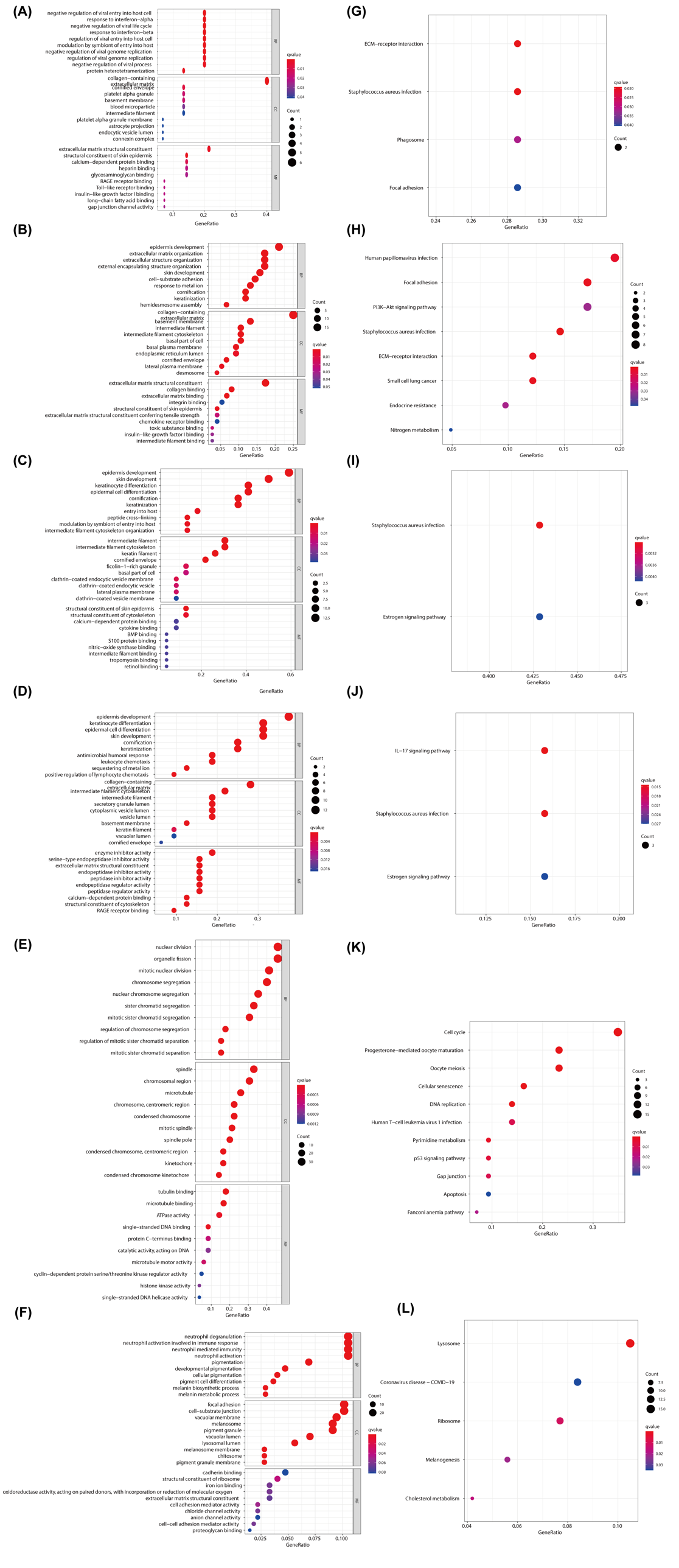

Supplement: Supplementary file 3 [file Image4.TIF]

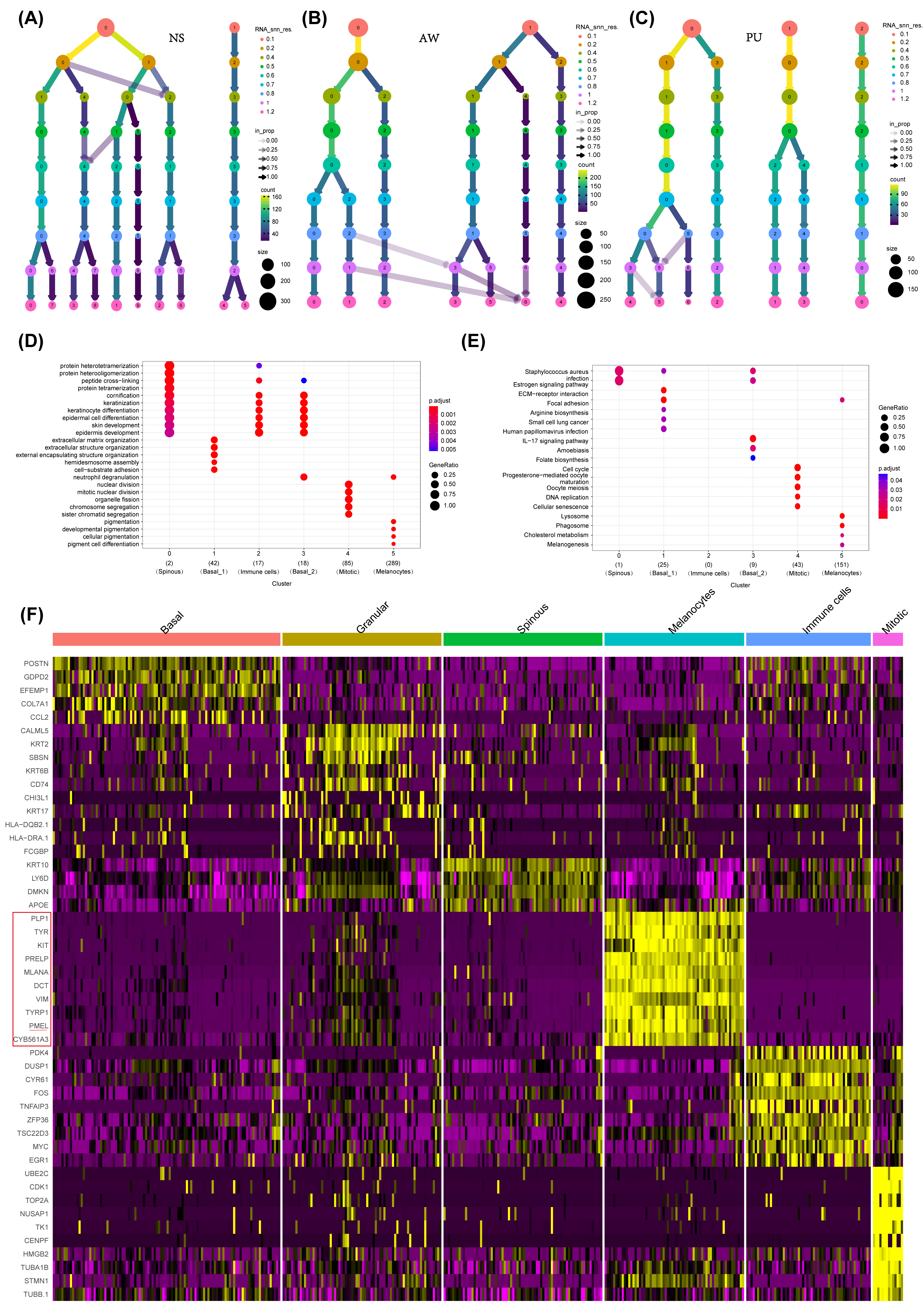

Supplement: Supplementary file 4 [file Image1.TIF]

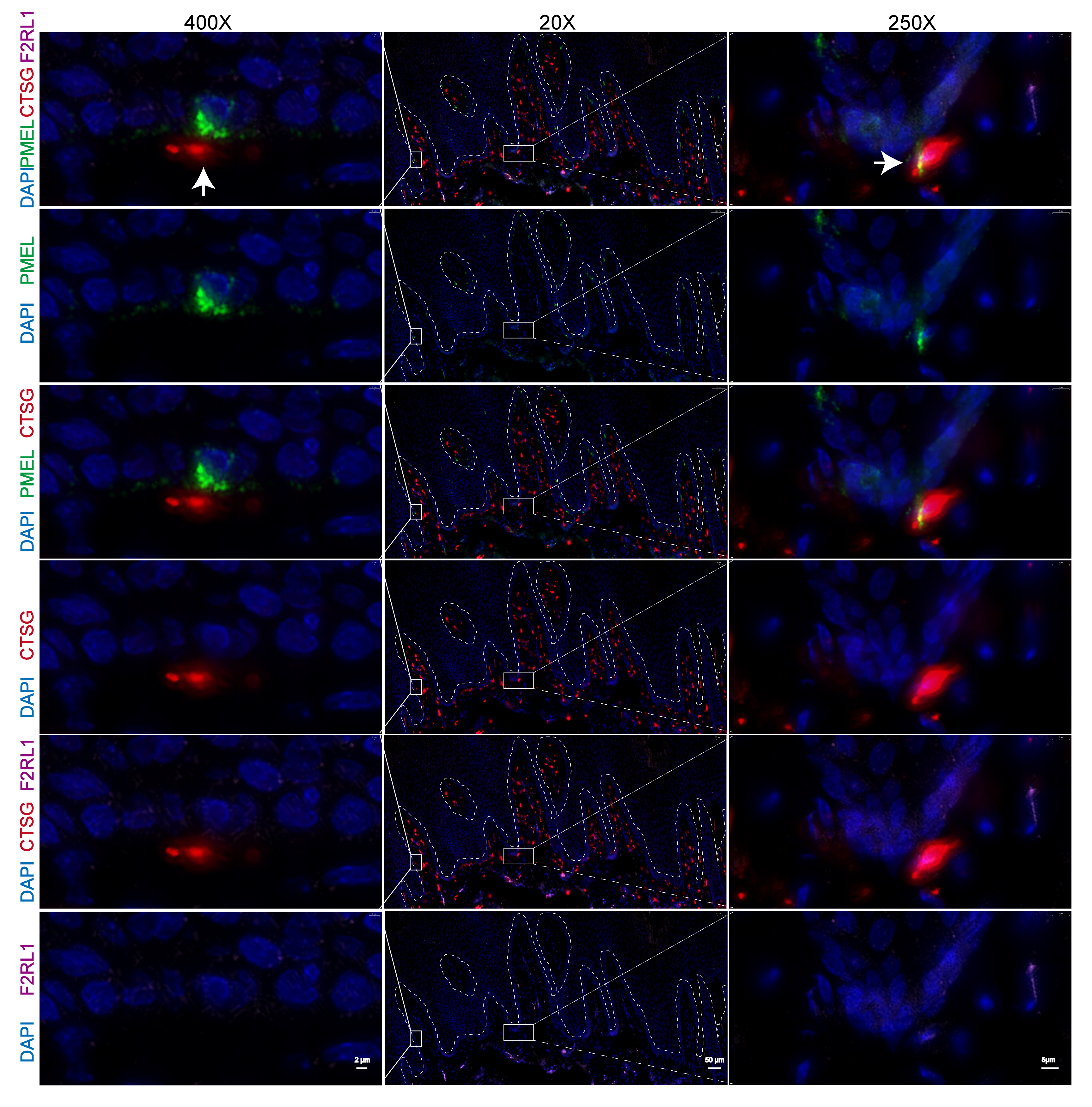

Supplement: Supplementary file 7 [file Image8.TIF]

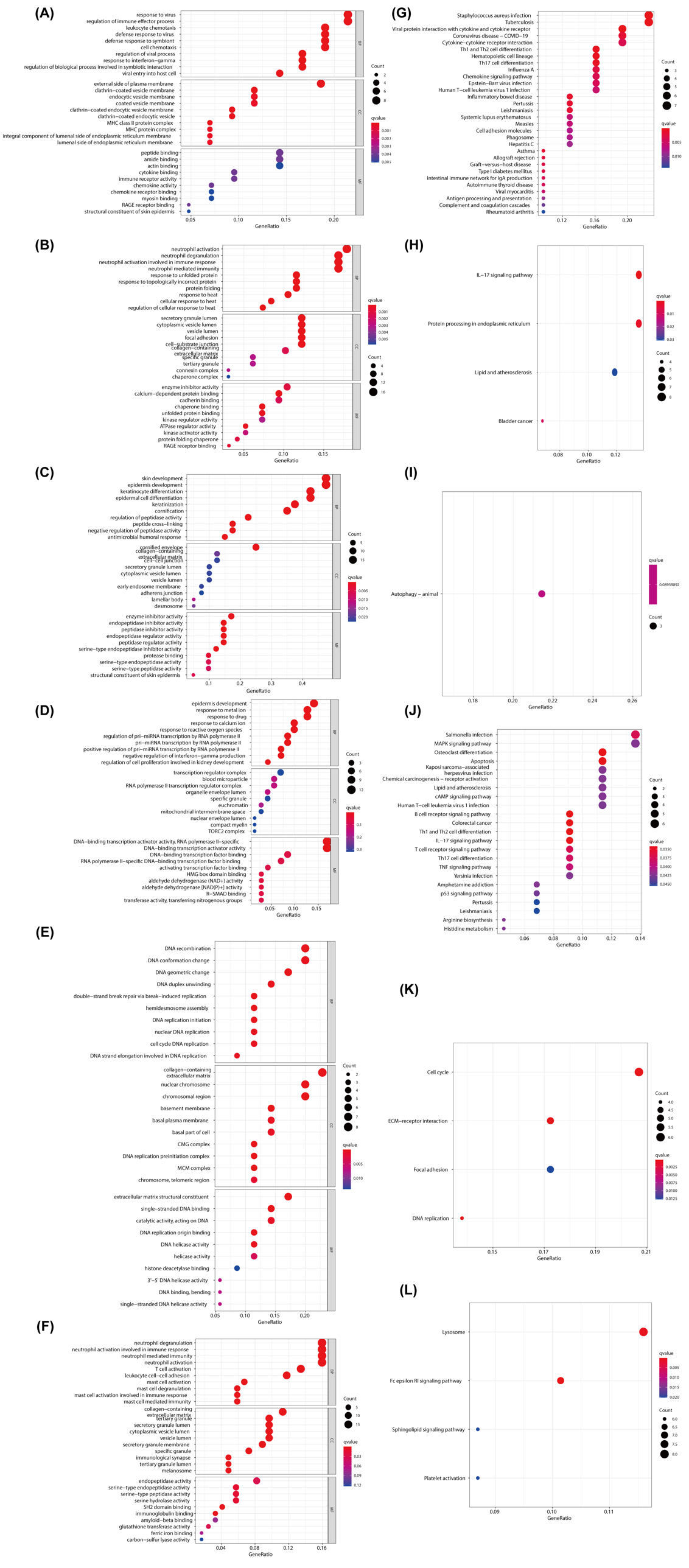

Supplement: Supplementary file 8 [file Image5.TIF]
