# Supplementary material for: Single-cell analysis reveals melanocytes may promote inflammation in chronic wounds through cathepsin G
Source: Front Genet. 2023 Jan 23;14:1072995. doi: 10.3389/fgene.2023.1072995 (PMC9900029; doi:10.3389/fgene.2023.1072995)
Supplement: Supplementary file 5 [file Table2.docx]

**Single-cell analysis reveals melanocytes may promote inflammation in chronic wounds through Cathepsin G**

Aobuliaximu Yakupu ^1,2*^, Di Zhang^1,2*^, Haonan Guan^1,2^, Minfei Jiang^1,2^, Jiaoyun Dong^1,2^, Yiwen Niu^1,2^, Jiajun Tang^1,2^, Yingkai Liu^1,2^, Xian Ma^1,2 #^ and Shuliang Lu^1,2 #^

Supplementary Table 1. The information of donors whose tissues were used for single-cell sequencing in the original article (GSE137897).

| Code | Gender | Age | Race | Site of wound | Duration of  wound | Grade |
| --- | --- | --- | --- | --- | --- | --- |
| H1 | Male | 37 | Caucasian | Gluteal area | --- | --- |
| H2 | Male | 30 | Caucasian | Gluteal area | --- | --- |
| H3 | Male | 26 | Caucasian | Gluteal area | --- | --- |
| H4 | Male | 35 | Caucasian | Gluteal area | --- | --- |
| PU1 | Male | 36 | Caucasian | Trochanter majus | 2 years | IV PU |
| PU2 | Male | 46 | Caucasian | Groin & trochanter majus into hip joint | 2 years | IV PU |
| PU3 | Male | 27 | Caucasian | Tuber ischiadicum | 7 years | IV PU |
| PU4 | Male | 62 | Caucasian | Tuber ischiadicum | 2 years | IV PU |
| PU5 | Male | 49 | Caucasian | Tuber ischiadicum | 1,5 years | IV PU |

PU: pressure ulcers.

Supplementary Table 2. The information of donors whose tissues were used for validation in the current study.

| Code | Gender | Age | Race | Site of wound | Duration of  wound | Grade |
| --- | --- | --- | --- | --- | --- | --- |
| AW1 | Female | 85 | yellow race | Ankle | 17 days | —— |
| AW2 | Female | 65 | yellow race | lower leg | 5 days | —— |
| AW3 | Female | 27 | yellow race | lower leg | 13 days | —— |
| AW4 | Female | 53 | yellow race | lower leg | 19 days | —— |
| PU1 | Male | 51 | yellow race | Tuber ischiadicum | 9 years | IV PU |
| PU2 | Male | 80 | yellow race | Sacrococcygeal Region | 1 year | IV PU |
| PU3 | male | 73 | yellow race | Tuber ischiadicum | 2 year | IV PU |
| PU4 | Female | 61 | yellow race | Tuber ischiadicum | 1.5 year | IV PU |
| PU5 | Female | 83 | yellow race | Sacrococcygeal Region | 2year | IV PU |

AW: acute wounds; PU: pressure ulcers. (Tissue samples for AW were taken from wounds several days after injuries)

Supplementary Table 3. The information about the secondary antibodies

| Secondary antibodies | Producer | Code | Dilution |
| --- | --- | --- | --- |
| HRP-goat anti-rabbit IgG | Servicebio | GB23303 | 1:500 |
| Cy3-goat anti-rabbit IgG | Servicebio | GB21303 | 1:300 |
| Cy5-goat anti-mouse IgG | Servicebio | GB27301 | 1:600 |
| DAPI | Servicebio | G1012 |  |

the secondary antibodies used for multiplex immunofluorescence.
